# Congenital Ventricular Diverticulum

**DOI:** 10.3390/jcm12093153

**Published:** 2023-04-27

**Authors:** Carmelo Massimiliano Rao, Fabiana Lucà, Claudio Franzutti, Giuseppe Scappatura, Nicola Arcadi, Pasquale Fratto, Francesco Antonio Benedetto, Sandro Gelsomino

**Affiliations:** 1 Cardiology Department, Grande Ospedale Metropolitano Bianchi-Melacrino-Morelli, 89124 Reggio Calabria, Italy; 2 Complex Operative Unit Radiology, Grande Ospedale Metropolitano Bianchi-Melacrino-Morelli, 89124 Reggio Calabria, Italy; 3 Cardio Thoraco Vascular Department, Cardiac Center, Great Metropolitan Hospital “Bianchi Melacrino Morelli”, 89124 Reggio Calabria, Italy; 4 Department of Cardiothoracic Surgery, Cardiovascular Research Institute, Maastricht University, 6211 LK Maastrich, The Netherlands

**Keywords:** congenital ventricular diverticulum (CVD), congenital cardiac disorder, re-entrant ventricular tachycardias (VT), cardiac magnetic resonance imaging (MRI)

## Abstract

Herein, we describe a 54-year-old patient with a congenital ventricular diverticulum (CVD), referred to our emergency department for presyncope episodes and multiple re-entrant ventricular tachycardias (VT). Significantly, echocardiographic findings were not clear, and the diagnosis was made by cardiac magnetic resonance imaging (CMRI), which showed the presence of an apical accessory cavity connected to the ventricle and contracting synchronously. CMRI allowed the differential diagnosis with other outpouching cardiac defects. The patient underwent a subcutaneous implantable cardioverter defibrillator (S-ICD) implant and was referred for heart transplantation (HT). The diagnosis, treatment, and main findings of the CVD are discussed in this case report.

## 1. Introduction

Congenital ventricular diverticulum (CVD) is a rare finding, and it has been reported as 0.05% of all congenital heart defects [1]. CVD is described as congenital outpouchings which contain all three layers of the cardiac wall (endocardium, myocardium, and pericardium) with a diameter ranging from 0.5 cm to 8–9 cm [2]. The diverticulum can be diagnosed during childhood when associated with other congenital cardiac abnormalities [3], but very often, it is clinically silent, and it may lead to life-threatening complications such as systemic embolisms, arrhythmias, heart failure (HF), and rupture [3].

Echocardiography is considered the gold standard for the diagnosis of CVD. Nonetheless, the differential diagnosis between CVD and other outpouchings, such as congenital ventricular aneurysm (CVA) and double-chambered ventricle (DCV), very important for treatment decisions, may be very challenging in echocardiography.

We present a case of a misdiagnosed CVD detected by cardiac magnetic resonance imaging (CMRI) after multiple episodes of re-entrant ventricular arrhythmias (VA) and discuss the differential diagnosis, treatment, and main findings of the disease.

## 2. Case Description

A 54-year-old male was referred to our emergency department for presyncope episodes. The patient was being treated for idiopathic dilated cardiomyopathy. He had no previous arrhythmic episodes, and his familiar history was negative. Four years before, the patient had suffered from a minor stroke with full neurological recovery. His past medical history showed statin-treated hypercholesterolemia and non-insulin-dependent diabetes. Pharmacological therapy included angiotensin receptor-neprilysin inhibitors (ARNIs), diuretics, and beta-blockers.

**Figure 1:**
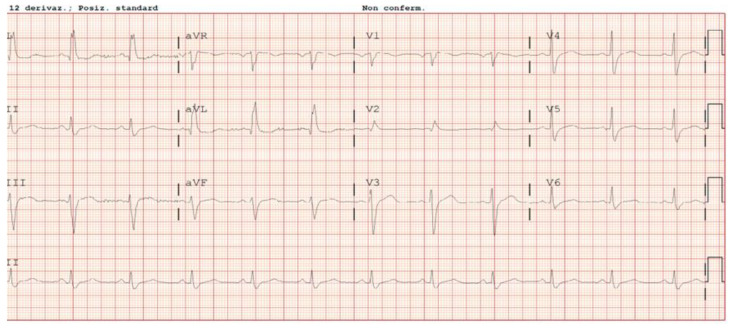
Electrocardiogram (ECG) shows sinus rhythm (SR) 75/min, initial pattern of left systolic ventricular overload.

During hospitalization, the patient had a prevalent sinus rhythm (SR) (Figure 1).

Natriuretic peptide levels were as follows: Brain Natriuretic Peptide (BNP), 2800 pg/mL; N-terminal pro-b-type natriuretic peptide (NT pro-BNP), 980 pg/mL. In addition, hypokalemia was detected (2.8 meq/L) and treated with mineralocorticoid receptor antagonists (MRA). The highly sensitive troponin I was normal.

**Figure 2:**
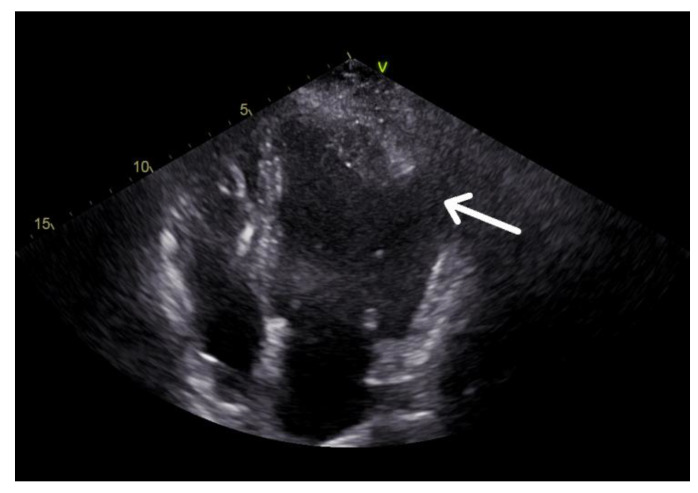
Two-dimensional transthoracic echocardiography (TTE) of a 4-chamber apical view shows an enlarged hypokinetic left ventricle. At the level of the apex, there was something resembling a left double-chambered ventricle (DCV, arrow).

At two-dimensional transthoracic echocardiography (TTE), the left ventricle was a hypokinetic left ventricle with a dual chamber-like feature (Figure 2).

**Figure 3:**
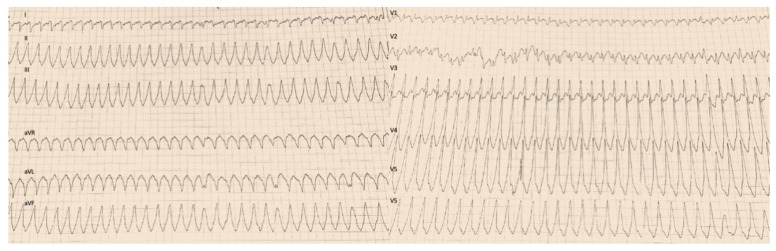
Electrocardiogram (ECG) shows a Ventricular Tachycardia, with intrinsicoid deflection delay > 60 msec. wide QRS, andfusion complexes.

The following day, a new presyncope episode occurred with ECG evidence of re-entrant ventricular tachycardia (VT) (Figure 3), which was electrically converted with External electrical cardioversion (EVC) because of hypotension and clinical instability.

**Figure 4:**
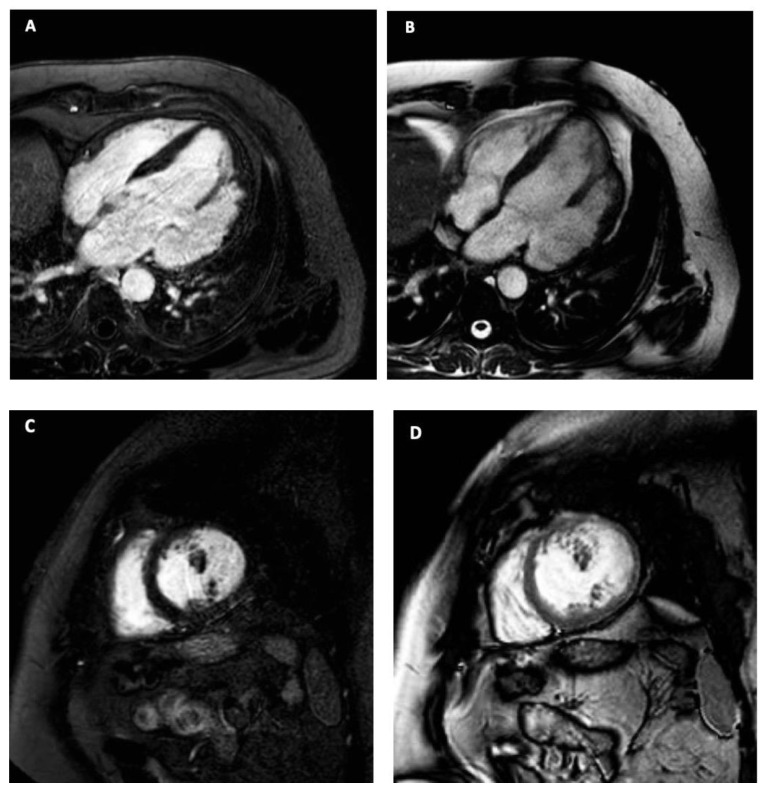
MRI findings. Long-axis 4-chamber LGE (**A**) and Cine Steady-state free-precession (SSFP) (**B**). Additionally, short-axis (**C**) LGE and (**D**) SSFP images show an apical accessory cavity connected to the ventricle in the absence of a thrombus or communication with the right ventricle.

Cardiac Magnetic Resonance (CMRI) showed the presence of an apical accessory cavity connected to the ventricle, which contracted in synchrony with the heart chambers (Figure 4). CMRI parameters are shown in Table 1.

No thrombus or communication evidence with the right ventricle (RV) was demonstrated. A diagnosis of CVD was made. 

Coronary angiography excluded significant obstructive coronary artery disease (CAD).

The patient underwent an S-ICD(EMBLEM MRI S-ICD).

The Heart Team opted for a conservative approach with clinical follow-up (FU), which was decided because of the small size of the cavity, the absence of a thrombus, and the absence of communication with the right ventricle (RV). Nonetheless, the patient developed symptoms despite full medical therapy. Therefore, the patient was put on the HT waiting list.

Congenital ventricular diverticulum (CVD) is a congenital disease often associated with other defects [4], as seen in the spectrum of Cantrell’s pentalogy, a congenital anomaly involving a midline anterior abdominal wall defect, a distal sternal cleft, a defect of the anterior diaphragm, and a defect of the apical pericardium with pericardial–peritoneal communication [5,6].

According to its localization in the ventricle [7], CVD can be apical or non-apical [1,2,3,8]. Apical CVD is a fingerlike contractile pouch with a narrow connection that contracts synchronously with the ventricle. In contrast, non-apical CVD is a large contractile pouch with a wide connection to the ventricle and is often not associated with other intracardiac abnormalities [3]. Based on the prevalence of myocardial fibers, CVD can be classified as muscular or fibrous, the former with synchronous contractility and the latter with dyskinetic or akinetic contraction [9].

Echocardiography plays a crucial role in the diagnostic workup. The diagnosis becomes more challenging in adults [3] when the defect is in “silent” zones, such as the anterior-basal and apical regions [10]. In addition, CVDs are often very difficult to distinguish from a congenital ventricular aneurysm (CVA) or double-chambered ventricle (DCV) by echocardiography. 

CVA is a predominantly fibrous large saccular akinetic or dyskinetic structure with a wide connection to the ventricle, and it does not include organized myocardium fibers [3,8,9]. DCV is a ventricular outpouching that contains all the cardiac layers and is characterized by a wide communicating neck to the ventricular chamber [7,10,11]. Nonetheless, the presence of endocardial fibroelastosis makes the DCV non-contractile [12]. 

Despite CVD having a better prognosis and being asymptomatic [3,7], CVA and DCV both carry a bad prognosis [3,13]. Therefore, a prompt differential diagnosis is mandatory in the presence of ventricular outpouching.

In our case, CDV was misdiagnosed as papillary muscle hypertrophy and double-chamber ventricle at echocardiography. At cine MRI, the synchronous contractility, the absence of fibroelastosis, and the narrow connection to the ventricle allowed us to exclude a DCV. Furthermore, the presence of three myocardial layers in the outpouching allowed us to exclude a CVA.

When a diagnosis of CDV is made, next-step modality imaging, such as CRMI Computed Tomography (CT) or cardiac catheterization, is employed to confirm the diagnosis and acquire more detailed information [14,15]. We believe that a timely CMRI should be considered when a ventricular protruding pouch or a double-chamber ventricle is suspected by echocardiography, even when the diagnosis is apparently clear. 

In contrast with CVA and DCV, in which surgery must also be considered in asymptomatic patients because of complications [3,11], the treatment of isolated, asymptomatic CVD is controversial [16,17].

Some authors suggest surgery when the diverticulum is in the proximity of the coronary arteries and causes chest pain, when there is the risk of impelling rupture or a high risk of life-threatening VA and HF in the absence of other contraindications, or when cardiac surgery is indicated for concomitant diseases [18].

Surgery consists of the identification of the neck of the diverticulum and its closure with a Dacron patch, followed by resection of the base of the diverticulum and closure of the diverticulum cavity with a Dacron or a pericardium patch (“sandwich method”) [18,19].

We adopted conservative management in this patient since the risk of postoperative HF related to the resection of the diverticular contracting myocardium wall would have been too high compared to the residual risk after controlling arrhythmias. In addition, this decision was also related due to the young age of the patient, his preference, and the possibility of putting him on the heart transplant waiting list. 

A life-saving S-ICD was placed. The transvenous placement was not chosen to avoid early complications related to lead insertion and long-term complications, such as lead endocarditis and lead dysfunction, considering there were neither anti-tachycardia pacing (ATP) nor permanent cardiac pacing indications. Another reason was to avoid the endovascular ICD implantation procedure’s possible complications.

## 3. Conclusions

MRI is highly recommended in asymptomatic patients in cases with the suspicion of CVD, allowing a prompt and accurate diagnosis to guide decision-making.

## Figures and Tables

**Table 1:** CMR Data.

Left Ventricle	
EDV	221 mL
EDVI	122 mL/m^2^
ESV	139 mL
ESVI	77 mL/m^2^
LVEF	37%
SV	82 mL
SVI	42 mL/m^2^
CO	6.31 L/min
CI	3.5 L/m^2^/min
EDLVST	10 mm
EDLWT	10 mm
LVM	108 g
LVMI	60 g/m^2^
**Right Ventricle**	
EDV	152 mL
EDVI	84 mL/m^2^
ESV	65 mL
ESVI	36 mL/m^2^
LVEF	57%
SV	87 mL
SVI	48 mL/m^2^

## Data Availability

Linkedin, Research Gate. Twitter, Instagram.

## Abbreviations

CMR, Cardiac Magnetic Resonance; EDV, End Diastolic Volume; EDVI, End Diastolic Volume Indexed; ESV, End Systolic Volume; ESVI, End Systolic Volume Indexed; LVEF, Left Ventricular Ejection Fraction; SV, Stroke Volume; SVI Stroke Volume Indexed; CO, Cardiac Output; CI, Cardiac Index; EDLVST, End Diastolic Left Ventricular Septal Thickness; EDLVWT, End Diastolic Left Ventricular Wall Thickness (inferior-lateral wall); LVM, Left Ventricular Mass; LVMI, Left Ventricular Mass Index.
